# Supplementary material for: The mechanism of aerobic exercise in ameliorating glycolipid metabolic disorders in metabolic syndrome rats via the miR-27a-PPARγ pathway
Source: PLoS One. 2026 Feb 11;21(2):e0341129. doi: 10.1371/journal.pone.0341129 (PMC12893564; doi:10.1371/journal.pone.0341129)
Supplement: Attachment — Submitted filename: Paper reveiw Overall Assessment.docx [file pone.0341129.s001.docx]

**Overall Assessment**

The study provides compelling evidence that aerobic exercise ameliorates metabolic syndrome (MS) via the miR-27a-PPARγ pathway, offering a novel mechanistic target for intervention. Well-designed experiments with appropriate controls, though some technical limitations weaken robustness. First to link exercise-induced metabolic benefits to miR-27a downregulation in MS, advancing the field beyond correlative studies.

**Weaknesses & Limitations**

**Technical Concerns**:

- **Sample Size Issues**: Underpowered analyses (e.g., LCAD mRNA trends, Page 19) risk Type II errors. *Power analysis is missing.*
- **Blot Integrity**: Cutting blots pre-hybridization (Page 12) is problematic; full blots should be supplemental.
- **Viral Delivery**: No data on knockdown efficiency over time (Page 39: "expression gradually decreased").

**Interpretational Gaps**:

- **miR-27a Regulation**: How exercise suppresses miR-27a (transcriptional vs. post-transcriptional?) is unexplored.
- **Human Relevance**: No discussion of miR-27a/PPARγ conservation in human MS or exercise responses.

**Presentation**:

- **Figure Clarity**: Some panels (e.g., Fig 1A–B vs. C–H) are poorly labeled; HE stains lack scale bars (Fig 5).
- **Repetition**: Methods for PCR/WB are overly detailed; streamline or supplement.

**Recommendations for Revision**

1. **Address Technical Gaps**:
   - Justify sample sizes with power calculations.
   - Include uncut blots in supplements.
   - Quantify miR-27a knockdown efficiency longitudinally.
2. **Expand Discussion**:
   - Compare findings to human miRNA studies (e.g., circulating miR-27a in MS patients).
   - Hypothesize mechanisms linking exercise to miR-27a downregulation (e.g., exosomal sorting?).
3. **Improve Clarity**:
   - Simplify abstract for broader accessibility.
   - Standardized figure labels (e.g., "Panel A" vs. "Fig 1A").

**Comparison to Field**

- **Advances Beyond Prior Work**:
  - Extends *Yu et al. (2018)* by showing PPARγ upregulation (not just IRS-1 inhibition) mediates exercise benefits.
  - Confirms *Chen T et al. (2019)* in vivo but adds exercise as a physiological modulator.
- **Unresolved Questions**:
  - Does miR-27a suppression also improve inflammation/fibrosis in MS?
