# Supplementary material for: The mechanism of aerobic exercise in ameliorating glycolipid metabolic disorders in metabolic syndrome rats via the miR-27a-PPARγ pathway
Source: PLoS One. 2026 Feb 11;21(2):e0341129. doi: 10.1371/journal.pone.0341129 (PMC12893564; doi:10.1371/journal.pone.0341129)
Supplement: Attachment — Submitted filename: Response to Reviewers.docx [file pone.0341129.s003.docx]

Dear editor and reviewers:

Thank you very much for the valuable and constructive comments on our manuscript (PONE-D-25-28234: The Mechanism of Aerobic Exercise in Ameliorating Glycolipid Metabolic Disorders in Metabolic Syndrome Rats via the miR-27a-PPARγ Pathway

). We appreciate the opportunity to improve the paper and responses to reviewers are addressed below. All changes made in the revised manuscript were highlighted in

yellow to make them easily identified.

**Journal Requirements**

1. Please ensure that your manuscript meets PLOS ONE's style requirements, including those for file naming. The PLOS ONE style templates can be found at

https://journals.plos.org/plosone/s/file?id=wjVg/PLOSOne_formatting_sample_main_body.pdf and

https://journals.plos.org/plosone/s/file?id=ba62/PLOSOne_formatting_sample_title_authors_affiliations.pdf

2. PLOS ONE now requires that authors provide the original uncropped and unadjusted images underlying all blot or gel results reported in a submission’s figures or Supporting Information files. This policy and the journal’s other requirements for blot/gel reporting and figure preparation are described in detail at https://journals.plos.org/plosone/s/figures#loc-blot-and-gel-reporting-requirements and https://journals.plos.org/plosone/s/figures#loc-preparing-figures-from-image-files. When you submit your revised manuscript, please ensure that your figures adhere fully to these guidelines and provide the original underlying images for all blot or gel data reported in your submission. See the following link for instructions on providing the original image data: https://journals.plos.org/plosone/s/figures#loc-original-images-for-blots-and-gels.

In your cover letter, please note whether your blot/gel image data are in Supporting Information or posted at a public data repository, provide the repository URL if relevant, and provide specific details as to which raw blot/gel images, if any, are not available. Email us at plosone@plos.org if you have any questions.

3. To comply with PLOS ONE submissions requirements, in your Methods section, please provide additional information regarding the experiments involving animals and ensure you have included details on (1) methods of sacrifice, and (2) efforts to alleviate suffering.

4. PLOS requires an ORCID iD for the corresponding author in Editorial Manager on papers submitted after December 6th, 2016. Please ensure that you have an ORCID iD and that it is validated in Editorial Manager. To do this, go to ‘Update my Information’ (in the upper left-hand corner of the main menu), and click on the Fetch/Validate link next to the ORCID field. This will take you to the ORCID site and allow you to create a new iD or authenticate a pre-existing iD in Editorial Manager.

5. We note that the grant information you provided in the ‘Funding Information’ and ‘Financial Disclosure’ sections do not match.

When you resubmit, please ensure that you provide the correct grant numbers for the awards you received for your study in the ‘Funding Information’ section.

6. We note that your Data Availability Statement is currently as follows: All relevant data are within the manuscript and its Supporting Information files.

Please confirm at this time whether or not your submission contains all raw data required to replicate the results of your study. Authors must share the “minimal data set” for their submission. PLOS defines the minimal data set to consist of the data required to replicate all study findings reported in the article, as well as related metadata and methods (https://journals.plos.org/plosone/s/data-availability#loc-minimal-data-set-definition).

For example, authors should submit the following data:

- The values behind the means, standard deviations and other measures reported;

- The values used to build graphs;

- The points extracted from images for analysis.

Authors do not need to submit their entire data set if only a portion of the data was used in the reported study.

If your submission does not contain these data, please either upload them as Supporting Information files or deposit them to a stable, public repository and provide us with the relevant URLs, DOIs, or accession numbers. For a list of recommended repositories, please see https://journals.plos.org/plosone/s/recommended-repositories.

If there are ethical or legal restrictions on sharing a de-identified data set, please explain them in detail (e.g., data contain potentially sensitive information, data are owned by a third-party organization, etc.) and who has imposed them (e.g., an ethics committee). Please also provide contact information for a data access committee, ethics committee, or other institutional body to which data requests may be sent. If data are owned by a third party, please indicate how others may request data access.

7. If the reviewer comments include a recommendation to cite specific previously published works, please review and evaluate these publications to determine whether they are relevant and should be cited. There is no requirement to cite these works unless the editor has indicated otherwise.

**Response:**

Thank you for your comments. We have supplemented and revised the manuscript according to your journal’s additional requirements, including formatting, original gel blot images (See attached supplementary information - Original gel blot images), details regarding laboratory animals (**Line 64-66, 79-82, 96-98，106-109**), verification of the corresponding author’s ORCID iD, verification and standardization of the correct funding number (In the Additional Information section of the submission system, we did not see an option for funding disclosure. We entered the funding information in the Manuscript Data - Funding Information section, but the submitted version displayed “The author(s) received no specific funding for this work.” This has left us confused), and confirmation of the data availability statement. These changes are highlighted in yellow in the corresponding sections of the manuscript. We hope this round of revision will make you satisfied and progress to the process.

**Overall Assessment**

The study provides compelling evidence that aerobic exercise ameliorates metabolic syndrome (MS) via the miR-27a-PPARγ pathway, offering a novel mechanistic target for intervention. Well-designed experiments with appropriate controls, though some technical limitations weaken robustness. First to link exercise-induced metabolic benefits to miR-27a downregulation in MS, advancing the field beyond correlative studies.

**Weaknesses & Limitations**

**Technical Concerns**:

- **Sample Size Issues**: Underpowered analyses (e.g., LCAD mRNA trends, Page 19) risk Type II errors. *Power analysis is missing.*
- **Blot Integrity**: Cutting blots pre-hybridization (Page 12) is problematic; full blots should be supplemental.
- **Viral Delivery**: No data on knockdown efficiency over time (Page 39: “expression gradually decreased”).

**Interpretational Gaps**:

- **miR-27a Regulation**: How exercise suppresses miR-27a (transcriptional vs. post-transcriptional?) is unexplored.
- **Human Relevance**: No discussion of miR-27a/PPARγ conservation in human MS or exercise responses.

**Presentation**:

- **Figure Clarity**: Some panels (e.g., Fig 1A–B vs. C–H) are poorly labeled; HE stains lack scale bars (Fig 5).
- **Repetition**: Methods for PCR/WB are overly detailed; streamline or supplement.

**Recommendations for Revision**

1. **Address Technical Gaps**:
   - Justify sample sizes with power calculations.
   - Include uncut blots in supplements.
   - Quantify miR-27a knockdown efficiency longitudinally.
2. **Expand Discussion**:
   - Compare findings to human miRNA studies (e.g., circulating miR-27a in MS patients).
   - Hypothesize mechanisms linking exercise to miR-27a downregulation (e.g., exosomal sorting?).
3. **Improve Clarity**:
   - Simplify abstract for broader accessibility.
   - Standardized figure labels (e.g., “Panel A” vs. “Fig 1A”).

**Comparison to Field**

- **Advances Beyond Prior Work**:
  - Extends *Yu et al. (2018)* by showing PPARγ upregulation (not just IRS-1 inhibition) mediates exercise benefits.
  - Confirms *Chen T et al. (2019)* in vivo but adds exercise as a physiological modulator.
- **Unresolved Questions**:
  - Does miR-27a suppression also improve inflammation/fibrosis in MS?

**Response:**

Thank you for your constructive comments and advice, which will significantly enhance the rigor and readability of this study. The revisions are outlined below:

1. **Technical gaps:**

• Power calculation: Thank you for your valuable comments. We fully recognize the importance of a priori power analysis. As this study did not involve pilot experiments, we were unable to perform such an analysis in advance, which we acknowledge as a limitation of our study design.

First, we supplemented the methodology section with the principles for determining the sample size for this study (Methods-Animals, diets and interventions. **Lines 83-93**) .

***The revised part:*** *The sample size in this study was determined based on the following principles: The sample size in this study was determined based on the following principles: (1) To account for individual variability and the risk of failure in establishing the high-fat and high-sugar diet-induced model, more animals were initially included in the modeling group to ensure that a sufficient number of successful model animals would be available for subsequent grouping; (2) In accordance with the ARRIVE 2.0 guidelines [18], when pilot data are unavailable, referencing existing literature is a valid approach for determining sample size. Studies in the field of metabolism commonly employ sample sizes of 5–8 animals per group, which has been proven effective in detecting statistically significant differences in key metabolic parameters [19, 20]; (3) Strict adherence to the 3R principles was maintained by controlling for factors such as animal strain, sex, age, and rearing conditions to minimize within-group variability and reduce animal usage.* **(Lines 83-93**)

Besides, we conducted a post-hoc power analysis following your suggestion. The results showed that for primary positive outcomes, such as the expression level of the core indicator miR-27a, the effect size was large (Cohen’s d > 4.9), indicating that the current sample size is sufficient to support these conclusions. We openly acknowledge that for comparisons with smaller effect sizes or non-significant results, the statistical power may be insufficient (Power < 0.8), implying a risk of Type II errors. We have clearly stated this limitation in the “Discussion” sections of the manuscript, interpreted all results more cautiously, and highlighted the need for verification in larger future studies (**Lines 738-747**). The relatively small sample size was a result of balancing animal ethics (the 3R principles) and the practical challenges of the experimental complexity (attrition due to viral modeling and exercise interventions). We are confident that, under these constraints, the data obtained are robust enough to address the main scientific questions of this study.

***The revised part:*** *However, several limitations must be acknowledged. First, we did not examine whether miR-27a suppression improves inflammation or fibrosis in MS, which are critical to disease progression. Second, potential synergistic effects involving other miRNAs in the regulatory network cannot be ruled out. Third, although effect sizes were observed for primary outcomes, the sample size may limit power to detect smaller effects or complex interactions, increasing the risk of Type II errors. Consequently, caution is warranted when interpreting negative outcomes, and larger studies are needed to confirm these findings. Future research should use single-cell sequencing to clarify tissue-specific mechanisms and explore combined exercise-genetic strategies for improved clinical translation.* (**Lines 738-747**)

• Uncut blots: The complete images for all western blots are provided in Supplementary information - Original gel blot images.

• Quantify miR-27a knockdown efficiency longitudinally: We appreciate the reviewer’s insightful comment regarding the longitudinal assessment of miR-27a knockdown efficiency. In the present study, due to technical limitations and animal welfare considerations (the 3R principle), we evaluated the knockdown efficacy at the experimental endpoint by measuring miR-27a expression in both skeletal muscle and liver tissues across all groups. While longitudinal measurements could provide additional kinetic information, our terminal assessment robustly confirmed significant miR-27a reduction in both silencing groups (SD-S-I and MS-S-I) compared with their respective null controls (SD-S-N and MS-S-N, *P* < 0.05), supporting the conclusion that effective and stable knockdown was achieved throughout the critical intervention period. Future studies employing longitudinal sampling or multiple time points would be valuable to further characterize the time-dependent dynamics of miR-27a suppression and its functional consequences.

2. **Discussion expansion**

• Human context and Mechanistic hypothesis: We thank the reviewer for this valuable suggestion. In response, we have expanded the Discussion section to include a comparison with human miRNA studies and have proposed a potential mechanism linking exercise to miR-27a downregulation. Specifically, we added clinical evidence showing elevated serum miR-27a levels in patients with metabolic syndrome and type 2 diabetes, and introduced exosome-mediated sorting and secretion as a plausible mechanism through which aerobic exercise may reduce miR-27a content (**Lines 552-555, Lines 577-590**).

3. **Clarity improvements**

We have simplified the abstract for enhanced readability (**Lines 2-11**) and standardized figure labels.

***The revised part:***

*Abstract: This study investigated how aerobic exercise improves metabolic disorders in rats with metabolic syndrome (MS) and explored the role of miR-27a in this process. MS was induced by a high-fat and high-sugar diet. After eight weeks of treadmill training, aerobic exercise was found to reduce metabolic abnormalities and decrease elevated miR-27a levels, while increasing the expression of PPARγ and key downstream molecules involved in glycolipid metabolism. Downregulating miR-27a via a viral vector produced benefits similar to those of exercise, and combining miR-27a inhibition with exercise led to further improvement. These results suggested that aerobic exercise alleviates MS-related metabolic disorders partly through suppressing miR-27a and promoting PPARγ signaling, revealing a potential therapeutic target.* (**Lines 2-11**)

4. **Comparison to field & unresolved questions**

Regarding this recommendation, we have added a section on strengths and limitations at the end of the discussion section (**Lines 735-747**).

***The revised part:*** *A key strength of this study is the translation of previous in vitro results to an in vivo setting, confirming PPARγ upregulation-beyond IRS-1 inhibition-as a central mechanism, as reported by Yu et al. [14], and validating the regulatory function of miR-27a under physiological conditions, consistent with Chen T et al. [34]. However, several limitations must be acknowledged. First, we did not examine whether miR-27a suppression improves inflammation or fibrosis in MS, which are critical to disease progression. Second, potential synergistic effects involving other miRNAs in the regulatory network cannot be ruled out. Third, although effect sizes were observed for primary outcomes, the sample size may limit power to detect smaller effects or complex interactions, increasing the risk of Type II errors. Consequently, caution is warranted when interpreting negative outcomes, and larger studies are needed to confirm these findings. Future research should use single-cell sequencing to clarify tissue-specific mechanisms and explore combined exercise-genetic strategies for improved clinical translation.* (**Lines 735-747**)

**Review Comments to the Author**

Please use the space provided to explain your answers to the questions above. You may also include additional comments for the author, including concerns about dual publication, research ethics, or publication ethics. (Please upload your review as an attachment if it exceeds 20,000 characters)

**Reviewer 1**

The manuscript entitled “The Mechanism of Aerobic Exercise in Ameliorating Glycolipid Metabolic Disorders in Metabolic Syndrome Rats via the miR-27a–PPARγ Pathway” explores a relevant and timely topic. The study examines how aerobic exercise mitigates metabolic syndrome in rats, proposing that this effect is mediated via the miR-27a–PPARγ signaling pathway. Overall, the topic is of interest to the field and fits within the scope of PLOS ONE. However, several major concerns must be addressed before this manuscript can be considered for publication.

- First, the manuscript lacks the sample size. Several results are described as “not statistically significant due to small sample size,” but no information about group sizes is presented. Moreover, it is not clear how the authors analyze their results. It is crucial to identify in the figure legends the n as well as the statistical analyses utilized.

**Response:**

Thank you for your comments. In response to this feedback, we have now clearly stated the sample size (n) for each experimental group in both the main text and the figure legends. Additionally, the specific statistical methods used for analysis have been described in the Methods section and were also indicated in the table and figure legends as appropriate.

- Although the authors frequently refer to the Lee’s index as a surrogate for obesity, this measure alone is insufficient. It would greatly strengthen the study if actual fat depot weights (e.g., epididymal or retroperitoneal fat) were provided to support conclusions about adiposity. The values of Lee’s index in this article are 10x lower than those of the literature. Moreover, the changes observed for fasting blood glucose and Lee index are within the normal range, but it is stated that the animals have metabolic syndrome.

**Response:**

Thank you for your comments. Regarding the suggestion to supplement Lee's index with fat depot weights measurements, we fully agree that adiposity indicators are crucial for evaluating obesity in metabolic syndrome. As shown in Figure 4E, we measured and reported the weight of epididymal and perirenal fat pads. Besides, we fully agree that MS is a multifaceted disorder that cannot be defined by any single parameter. As described in the Methods section, the MS model in this study was rigorously diagnosed based on the fulfillment of at least two of the established criteria, including elevated blood pressure, fasting blood glucose ≥5.6 mmol/L, dyslipidemia, and significantly increased body weight relative to controls. All model animals met these criteria before intervention. Regarding the Lee’s index value being an order of magnitude lower than those reported in some literature, this discrepancy is probably attributable to differences in calculation formulae. In our study, Lee’s index was computed using the classical formula: body weight (g)^0.33 / naso-anal length (cm) × 1000. Other studies may omit the multiplier (×1000) or use alternative units, leading to substantial differences in absolute values. It is also noteworthy that the change in Lee’s index alone did not reach significant difference after modeling in experimentation 2, which may reflect the short intervention period or the specific metabolic characteristics of this model. We acknowledge this as one of the study limitations. Future research will employ larger sample sizes, longer intervention periods, or more precise body composition analysis methods (such as dual-energy X-ray absorptiometry) to more reliably assess the impact of exercise on fat deposition within this model. We sincerely appreciate the reviewer’s feedback, which has helped us clarify these important methodological aspects.

- Why did the authors not show serum insulin levels?

**Response:**

Thank you for your comments. We did measure fasting serum insulin, but because HOMA-IR is derived from both fasting glucose and insulin it provides a single, integrated estimate of insulin resistance. To keep the figures concise we show only HOMA-IR, which we feel better captures the overall insulin-resistant phenotype in this model. If preferred, we will be happy to add separate panels for fasting insulin and glucose.

- There are also issues related to data presentation. It is very hard to understand the results from figure 4 to 6. I could not understand the comparison among groups that vary in the figures. Some figure legends lack detail, and blot images do not clearly show molecular weight markers or quantification methods. The figures should be revised to improve clarity, standardize units, and include proper labels. The text would benefit from language editing, as there are frequent redundancies, awkward phrasing, and grammatical errors that impede readability. A professional English editing service is strongly recommended.

In conclusion, while this manuscript addresses an important question and employs a multifaceted experimental approach, it currently suffers from issues in experimental rigor, statistical interpretation, and clarity of presentation.

**Response:**

Thank you for your comments. We have carefully optimized Figures 4 to 6 as much as possible to improve data presentation, including adding detailed annotations for groupings in the figure captions and incorporating molecular weight markers in the blot images. Additionally, we have polished the manuscript’s language to enhance readability and correct grammatical errors.

**Reviewer 2**

The manuscript presents a technically robust study with well-designed experiments, appropriate controls, and sufficient replication. The data strongly support the stated conclusions. The statistical methods are rigorous and applied correctly, ensuring the reliability of the results. The authors have complied with PLOS ONE’s data policy, making all underlying data fully available (or specifying valid restrictions, if any). The manuscript is clearly written in standard English, with no major grammatical or typographical errors. Minor revisions (if any) should address only superficial language edits. Overall, the study meets PLOS ONE’s criteria for sound methodology, transparency, and clarity. No major revisions are required.

**Response:**

Thank you for your positive comments. We appreciate the time and effort you devoted to enhancing the quality of our study. In response to your suggestion, we have carefully polished the English throughout the manuscript to improve clarity and readability.
